# Commentary on Nagy et al. (2025): bestiary of questionable research practices in psychology

**DOI:** 10.3389/fpsyg.2026.1692416

**Published:** 2026-02-16

**Authors:** Pablo Ezequiel Flores-Kanter

**Affiliations:** 1 Universidad Siglo 21, Córdoba, Argentina; 2 Escuela de Posgrado, Universidad Continental, Lima, Perú

**Keywords:** ethical principles and values, misconduct, questionable research practices (QRPs), responsible research practices, taxonomy

Nagy et al. (2025) present an extensive and precise review of previous attempts to define Questionable Research Practices (QRPs) in psychology. Based on this review, they raise a series of concerns about current definitions and argue for the need to redefine these practices. Using an expert-consensus procedure, Nagy et al. (2025) arrive at a consensual definition of QRPs, identifying a total of 40 QRPs. In this commentary, I argue that (a) to achieve a more comprehensive and precise definition of QRPs, it is necessary to start from a clear ethical framework; and (b) there is a need to develop a taxonomic proposal which, based on specific ethical principles, allows for a more appropriate categorization of research practices.

Efforts such as those of Nagy et al. (2025) are relevant and, together with other previous contributions with similar intentions (Bouter et al., 2016; Chin et al., 2021; Ioannidis, 2005, 2012; John et al., 2012; Manapat et al., 2024; Munafò et al., 2017; Sacco et al., 2019; Simmons et al., 2011; Stefan and Schönbrodt, 2023; Steneck, 2006; Wigboldus and Dotsch, 2016), advance the definition, categorization, and identification of QRPs. This is extremely valuable, because a fundamental prerequisite for the adequate evaluation and intervention regarding these practices is their proper definition. However, I believe that current developments on this topic, including the contribution by Nagy et al. (2025), lack an explicit ethical framework in their formulation. The main objective of this commentary is to bring ethical perspective into the discussion on this topic, culminating in the proposal of a taxonomy grounded in three fundamental ethical principles: honesty, openness, and prudence (HOPE principles).

## 1 Definitions of questionable research practices

As stated at the outset, Nagy et al. (2025) provides an exhaustive and precise review of previous attempts to define QRPs, which I will not repeat here. However, I consider it important to begin with the consensual definition they provide in order to highlight the relevance of an ethical approach. Nagy et al. (2025) present the following definition of QRPs:

*Questionable research practices (QRPs) are ways of producing, maintaining, sharing, analyzing, or interpreting data that are likely to produce misleading conclusions, typically in the interest of the researcher*. ***QRPs are not normally considered to include research*** ***practices that are prohibited or proscribed in the researcher's field (e.g., fraud, research*** ***misconduct). Neither do they include random researcher error (e.g., data loss)*** (emphasis mine).

I have emphasized the second part of this definition because it makes more evident the pertinence of incorporating ethical arguments to achieve a more adequate definition of QRPs. On this point, the authors argue:

…* the definition excludes fraud and random errors as QRPs. We considered fraud, for example, the fabrication of data, clearly unacceptable and therefore not questionable. We also acknowledge that errors may happen in the research process for many reasons (Nagy et al.*, *2025**)*.

What remains unclear in this clarification is what ultimately determines the distinction between conduct such as fraud and questionable conduct. This would be clearer if the ethical principle(s) violated in each case were specified. The authors continue:

*While working on this manuscript, we had several discussions about the role of researchers' intentions in QRPs. Our view is that QRPs lie on a continuum between (but not including) fraud and random error. QRPs may be strongly motivated or simply based on a lack of knowledge or social pressure, and it is important not to assume that all engagement in QRPs is intentional. Ultimately, we decided to stay clear of judgments of intentionality and instead focus on providing a detailed description of the behaviors and their attributes, indicators, and preventive measures*.

However, if the authors started from explicit ethical arguments and principles, they could identify that intentionality is a key factor in distinguishing between fraudulent and questionable conduct. Furthermore, contrary to the position of Nagy et al. (2025), it would be possible to place “honest” mistakes or random errors within what can be considered QRPs. Below, I develop some ethical notions that I believe are central to the purpose of this commentary.

## 2 Research ethics: conceptual contributions to the evaluation of research behavior

It is possible to identify certain ethical standards (principle-rules) in scientific practice. These standards are based on two conceptual foundations: science and morality (Resnik, 2005; Shamoo and Resnik, 2009; Tijdink et al., 2021). Ethical conduct in science therefore involves (a) not violating commonly accepted moral standards and (b) promoting the advancement of scientific goals (such as the pursuit of knowledge and the resolution of practical problems). Both aspects are intimately linked in practice and cannot truly be separated (Bosma and Granger, 2022).

For example, fraud is unethical because it involves lying and deceiving, which is morally wrong, but also because fraudulent practices such as data fabrication (with the intention of deceiving) generate error and undermine trust in scientific results (Shamoo and Resnik, 2009). Here, I focus on three fundamental ethical principles—honesty, prudence, and openness (Bosma and Granger, 2022; Resnik, 2005; Shamoo and Resnik, 2009; Tijdink et al., 2021). I consider these principles central to current debates on the lack of credibility of science and the low reproducibility of scientific findings, and they enable a clearer conceptualization and taxonomy of scientific practice.

Honesty as an ethical principle indicates that scientists must not fabricate, falsify, or misrepresent data or results. Dishonesty involves the intent to deceive (by lying, concealing information, or misleading) an audience that expects to be told the truth (Resnik, 2005). The principle of prudence holds that researchers should avoid errors in research: they should minimize human and methodological errors, avoiding self-deception, bias, and conflicts of interest (Resnik, 2005). Lack of prudence differs from dishonesty in that it does not involve intent to deceive. While less serious than dishonesty from an ethical standpoint, such “honest” errors should still be avoided, as they can waste resources, erode trust, and lead to negative or even disastrous social consequences. These errors can thus be classified as negligent conduct.

Finally, the principle of openness indicates that scientists should share data, results, ideas, techniques, and materials (World Medical Association, 2013) in order to allow other researchers to review their work (Resnik, 2005, 2018). Scientists have a moral obligation to avoid concealment in order to help others (Bosma and Granger, 2022). More general ethical principles of justice and reciprocity also require researchers to contribute to the accumulation of scientific knowledge, which necessitates that research content be (re)usable. A key step toward these objectives is improving reporting practices. Poor reporting prevents the appropriate implementation of research results, which may expose individuals to unnecessary risks or deprive them of potential benefits lost through the improper use of results. Poor reporting can also lead to misuse or waste of resources allocated to reproduce findings. For all these reasons, (good) reporting practices are considered primarily a moral obligation (Aczel et al., 2019; Bosma and Granger, 2022; Moher, 2007; Needleman et al., 2008).

## 3 Research conduct: the HOPE taxonomy

**Figure 1:**
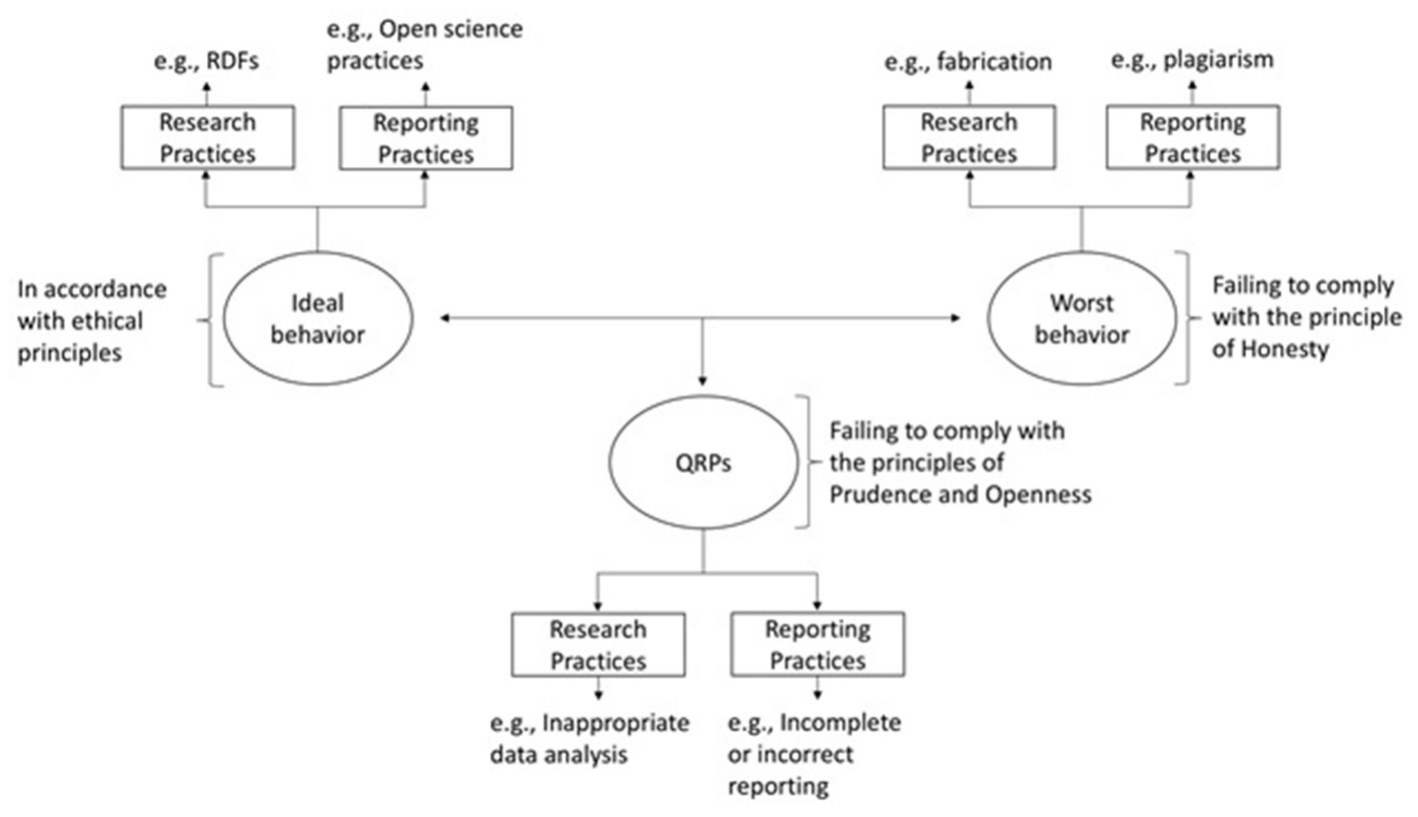
Taxonomy based on three principal research ethics principles (HOPE taxonomy).

The preceding ethical discussion allows for the development of a continuum differentiating research behaviors (see Figure 1): from (1) fraudulent or worst research behavior, which violates the ethical principle of honesty, to (2) ideal behavior, which involves responsible conduct of research in accordance with the three principal research ethics principles (HOPE principles). In between, we find (3) QRPs. These, while complying with the principle of honesty, do not fully align with the principles of prudence and openness.

*Figure 1*. RDF = researcher degree of freedom, as defined by Manapat et al. (2024).

This new taxonomy clarifies criteria for distinguishing research conduct, from ideal to worst behavior, and allows for the reclassification of certain practices. Fabrication, for example, is misconduct only when it involves intent to deceive; in contrast, data fabrication in transparent simulation studies for methodological purposes is responsible conduct. Similarly, reanalyzing data through multiple methods in search of statistically significant results (i.e., p-hacking) is questionable only if concealed; when justified and transparently reported (e.g., exploratory studies), it reflects responsible practice.

Applying the HOPE taxonomy, some practices listed by Nagy et al. (2025)—such as false authorship and PARKing—that clearly indicate dishonest intent should be classified as misconduct, rather than as questionable research practices. Other practices identified as QRPs by Nagy et al. (2025), however, could be categorized as responsible research practices, QRPs, or misconduct, depending on which HOPE ethical principle is violated—or upheld.

For example, Hypothesizing After the Results are Known (HARKing) is appropriately classified as a QRP in cases where the principle of openness is violated, such as when reports fail to clearly distinguish between exploratory and confirmatory components of a study. In contrast, the same practice may be considered a responsible research practice when hypotheses generated after observing the results are explicitly and transparently reported as such. However, if HARKing is not transparently disclosed and is used with the intention of concealing information or misleading the reader, it should clearly be classified as misconduct.

The HOPE taxonomy thus enables a more precise evaluation of research practices, particularly by clarifying what differentiates the various categories of practices. In this way, the HOPE taxonomy helps explain why the same practice (e.g., fabrication) may be classified differently depending on how it is conducted and reported. For instance, fabrication may constitute a responsible research practice when it adheres to the principles of honesty, openness, and prudence—such as when data simulation methods are explicitly described, fully reproducible, provide access to all relevant information, and follow current best-practice recommendations for the appropriate use and reporting of simulations.

In contrast, fabrication may be considered a QRP when it violates the principles of (a) openness and/or (b) prudence—for example, (a) when the description of the simulation method is imprecise or incomplete, or when the analytical syntax is not made available, thereby hindering reproducibility; or (b) when current best-practice guidelines for conducting simulations are not followed. Finally, fabrication constitutes misconduct when it violates the principle of honesty—such as when simulated data are not disclosed as such, and this omission is intended to deceive the reader.

## 4 Conclusions

The lack of consensus in definitions and proposed taxonomies for QRPs hinders proper analysis of our own and our peers' research practices. Among other consequences, this may generate unnecessary fears among researchers, as well as the abandonment of (good) practices that in some cases are misclassified as questionable (Wigboldus and Dotsch, 2016). With the proposed HOPE taxonomy, I have sought to contribute to works such as Nagy et al. (2025) with the aim of improving previous conceptualizations and proposals, placing ethics and its principles at the core of scientific practice.
